# Book review

**DOI:** 10.1054/bjoc.2000.1393

**Published:** 2000-10

**Authors:** D Lloyd

**Affiliations:** Medical Ethics, Centre of Medical Law and Ethics, King's College London, London, UK


*British Journal of Cancer* (2000) **83**(8), 1118–1119
© 2000 Cancer Research Campaign
doi: 10.1054/ bjoc.2000.1393, available online at http://www.idealibrary.com on IDEAL®

### Book review

### *Palliative Care Ethics: A Companion for all Specialties* (2nd edition)

Fiona Randall and R. S. Downie, pp 312 + xv,
Oxford University Press, 1999. Price £21.95
ISBN 0 19 2630687

There is much to admire in this book. It presents its material clearly, and strikes a good balance between accessibility and philosophical technicality. There is material included here on euthanasia, resource allocation, confidentiality, and the nature of clinical decision making, all of which would be of interest to health professionals in palliative care.

The introductory chapter sets out a broad picture of the domain of health care ethics, and unites this with a vision of the aims of palliative care. Subsequent chapters examine in detail the ethical significance of different aspects of palliative care, such as the patient–carer relationship, the relative–patient relationship, information giving, and the provision of emotional care. Each chapter ends with a helpful summary of its main points. The later chapters presuppose philosophical themes developed in the first, but otherwise they are largely independent.

Randall and Downie endorse the familiar ‘four principles’ widely regarded as of special importance for examining ethical problems in health care: beneficence; non-maleficence; respect for autonomy; and justice. Unlike some authors who adopt this approach to health care ethics, Randall and Downie clearly are alert to the conflicts and inconsistencies inherent in this set of principles. For example, a clash between beneficence and respect for patient autonomy occurs when a health professional seeks to benefit a patient by administering a particular treatment that the patient chooses to forego. When general principles collide like this, the particularities of any given situation determine the relative weight each one assumes. According to this approach to health care ethics, there is no absolutely dominant principle, no consistent hierarchy. Additional considerations are often drawn upon in an effort to establish priorities in specific cases.

To this end, Randall and Downie also briefly consider the contrast between the ‘four principles’ and the principle of utility – that one ought to do whatever will maximize the good for the greatest number of morally relevant beings – though they do not explore the conceptual difficulties raised by that contrast in this book. This is a little disappointing, given that they wish to add utility to their list of principles of health care ethics.

However, for Randall and Downie, ‘the underlying ethical concern … which is … the controlling moral component in all palliative care’ (p. 21) is the Aristotelian notion of *phronesis*, or practical wisdom. This is a capacity we all have to varying degrees, and it defies encapsulation in any kind of professional formalization. Rather, individuals who have developed *phronesis* manage to comprehend their accumulated life experience in a particularly integrated way, such that they can rely on it to form the basis of sound ethical judgement. Thus *phronesis*, being something that, so to speak, emerges from within, cannot be handed over from one person to another as a simple set of directives. But the appeal to *phronesis* introduces another question that the theoretical core of the book leaves unanswered: for those individuals who manage successfully to cultivate *phronesis* and be guided by it, what use are four, or five or (as in the case of this book) six principles of health care ethics?

Randall and Downie are happy to take a controversial stance where they think the weight of argument justifies it. There are two particular trends in palliative care they take particular issue with – the tendency to accord supremacy to patient autonomy, and the delivery of emotional care via the application of professional counselling and communication skills. In both of these respects they appear to be swimming against the tide. But the cases made in favour of their positions are well reasoned and compelling. In the case of patient autonomy, they correctly point out that this cannot be the leading ethical principle in palliative care, since it offers no guidance on the care of non-autonomous patients. They are also highly critical of consumer preference models of health care; patients will sometimes demand treatments that clinicians know will do them more harm than good, and it would be wrong for clinicians to compromise their commitment to not knowingly harm their patients.

As to emotional care, they contend that there is no such thing as professional expertise in this respect. They also maintain that professional counselling is an inappropriate tool to employ in the palliative care setting, arguing that formal counselling processes undermine the ordinary lines of human interaction that are required for successful emotional care to be delivered.

Randall and Downie’s position on a further controversial topic, the question of euthanasia, is not entirely clear. They concede that letting a person die – sometimes called ‘passive euthanasia’ – can be morally acceptable, because ‘the burdens and risks of life-prolonging or life-sustaining treatment outweight its benefits’ (p. 123). They also oppose active euthanasia largely on the grounds that ‘society needs to maintain its prohibition against killing (murder) in order to protect its members’ (p. 270). However, they do not make it clear why there is no parallel need for society to maintain a prohibition against letting people die in order to protect its members. And if the burdens and risks of life-prolonging or life-sustaining treatment are sufficient grounds on which to condone passive euthanasia, some readers will wonder why this does not provide a similarly compelling reason for recognizing the moral (if not legal) validity of competent requests for active euthanasia.

They offer something further on this issue in the final chapter of the book, ‘Quality and value of life’. The argument here is based on a distinction they introduce between ‘ethics’ and ‘values’. Values, as defined here, are our subjective preferences, and are hostages to fashion and whim. Ethics, by contrast, guides our conduct in ways that have a more consolidated intersubjective, if not universal, appeal; it is ‘a system of interpersonal rules for the better ordering of human life’ (p. 304). On the basis of this distinction they claim that the value of lives is independent of the question of whether any given life ought to be brought to a premature end. They hold that ‘no one has a right to do away with a life, or negligently to harm it, even if it is lacking in value’ (p. 305). Yet the precise way in which the distinction supports this important conclusion is not worked out in sufficient detail to be fully persuasive.

In spite of the unexplored philosophical difficulties at its theoretical heart, this book does a good job in providing a useful introduction to the application of systematic thinking about ethical problems facing the members of palliative care teams. Although written primarily with this group of health professionals in mind, many others will find that this book has useful things to say about the ethical dimension of their work. It would make a useful supplementary text for a variety of courses on health care ethics.

*Dr D Lloyd*
*Lecturer in Medical Ethics*
*Centre of Medical Law and Ethics*
*King’s College London*
*London, UK*

*© 2000 Cancer Research Campaign*

